# Diagnostic dilemma of Hodgkin’s lymphoma versus tuberculosis: a case report and review of the literature

**DOI:** 10.1186/s13256-021-02927-x

**Published:** 2021-07-19

**Authors:** Anamika Banerjee, Kaljit Bhuller, Rajini Sudhir, Amrita Bajaj

**Affiliations:** 1 Faculty of Medicine, Imperial College London, London, UK; 2 Haematology, Children’s and Adolescent Services, University Hospitals of Leicester NHS Trust, Leicester, UK; 3 Respiratory Medicine, University Hospitals of Leicester NHS Trust, Leicester, UK; 4 Radiology, University Hospitals of Leicester NHS Trust, Leicester, UK

**Keywords:** Case report, Literature review, Hodgkin’s lymphoma, Tuberculosis, Diagnostic delay, Guidelines

## Abstract

**Background:**

Hodgkin’s Lymphoma (HL) is a rare malignancy characterised histologically by the presence of Reed-Sternberg cells. Diagnosis of lymphomas can be difficult due to broad, non-specific presentations of disease, which can be similar to several other conditions ranging from infective, inflammatory or malignant causes, with one of the most common differentials being tuberculosis (TB). We aim to highlight the diagnostic dilemma of TB versus lymphoma with an atypical presentation of HL and explored areas for further research and improvement with a non-systematic literature review using MEDLINE database and Google Scholar. Written consent was obtained from the patient in compliance with ethical guidelines.

**Case presentation:**

A 23-year-old Asian female initially presented to rheumatology with over a one-year history of neuropathic pain, alongside abnormal white cell count and inflammatory markers. This was investigated with magnetic resonance imaging resulting in an incidental finding of mediastinal mass and pulmonary infiltrates. An initial diagnosis of TB was made despite testing negative for acid-fast bacilli and anti-tubercular treatment was commenced. Four months later, following clinical deterioration and further investigations, a mediastinal biopsy assisted in diagnosing Stage IV HL.

**Conclusions:**

Lymphoma is often misdiagnosed as TB, prolonging time to treatment and may adversely impact patient prognosis due to disease progression. Existing TB guidelines for smear-negative cases are not clear when to consider alternative diagnoses. In smear-negative TB, lymphoma should be considered as a differential and definitive diagnostic tests such as molecular testing and histological examination of biopsies should be considered earlier in the diagnostic work-up to prevent diagnostic delay.

## Introduction

Hodgkin’s lymphoma (HL) is a rare B-cell lymphoma, accounting for 1% of all new tumour diagnoses worldwide. It is characterised by the presence of Reed-Sternberg cells and accounts for approximately 15% of lymphomas (with 85% being non-Hodgkin) [1]. HL is more common in children and young adults, with the nodular sclerosis subtype of classical HL predominating in this age group [2].

Despite improved understanding of disease and advances in technology, lymphomas continue to present diagnostic challenges. The typical presentation of lymphomas includes palpable lymph nodes (most commonly in the neck) and B symptoms that is fever, night sweats and unexpected weight loss > 10% over the last 6 months [3]. However, lymphomas can have a wide variety of presentations especially in early-stage disease, resulting in diagnostic delay [4]. This applies to mediastinal and/or pulmonary HL, which are often misdiagnosed. One of the most common differentials is tuberculosis (TB).

Various studies and case reports have highlighted the difficulties of distinguishing between lymphoma and TB [5], resulting in diagnostic delay which may worsen patient prognosis. This is still a problem today. Furthermore, TB and HL may coexist, which is confounding and brings further complexities.

We discuss a case of an atypical presentation of classical HL and literature review to raise awareness of this diagnostic dilemma and explore areas of further investigation and research to reduce diagnostic delay.

## Case presentation

A 23-year-old Asian female presented to rheumatology in February 2020 following GP referral with history of worsening pain and paraesthesia of the right upper limb since 2018. She was managing with simple analgesics and physiotherapy under care of musculoskeletal and neurology team. Her symptoms were initially intermittent but became persistent from August 2019 and had progressed to involve the neck, upper-back and left arm. The severity of pain was disrupting her daily activities and sleep.

She was a non-smoker and non-drinker. There was no significant past medical history, except migraine.

She had a family history of Type 2 diabetes mellitus, hypertension and cancer, including thyroid and prostate cancer.

She was born in the UK to parents of Indian origin and had received the Bacillus Calmette-Guérin (BCG) vaccine at birth. She travelled to India every other year to visit relatives. She had no known contacts of TB.

Her weight was 54 kg, and body mass index (BMI) was 21. On examination, there was slightly reduced power noted in the right upper limb and myofascial tenderness over the cervical and thoracic spine, but range of motion and reflexes remained fully intact.

Initial blood tests demonstrated elevated white cell count (WCC) of 14.8 × 10^9^/L (normal range (NR) 4–11 × 10^9^/L) with neutrophilia (12.0 × 10^9^/L; NR 2–7.5 × 10^9^/L), elevated C-reactive protein(CRP) (31.4 mg/L; NR < 5 mg/L), elevated total protein (89 g/L; NR 60–83 g/L) with hypergammaglobulinaemia (49 g/L; NR 7–16 g/L), alongside low vitamin D (63.4 nmol/L; NR > 70nmol/L). Auto-antibody screen including anti-nuclear antibody, rheumatoid factor and anti-citrullinated protein antibodies were negative.

**Fig. 1:**
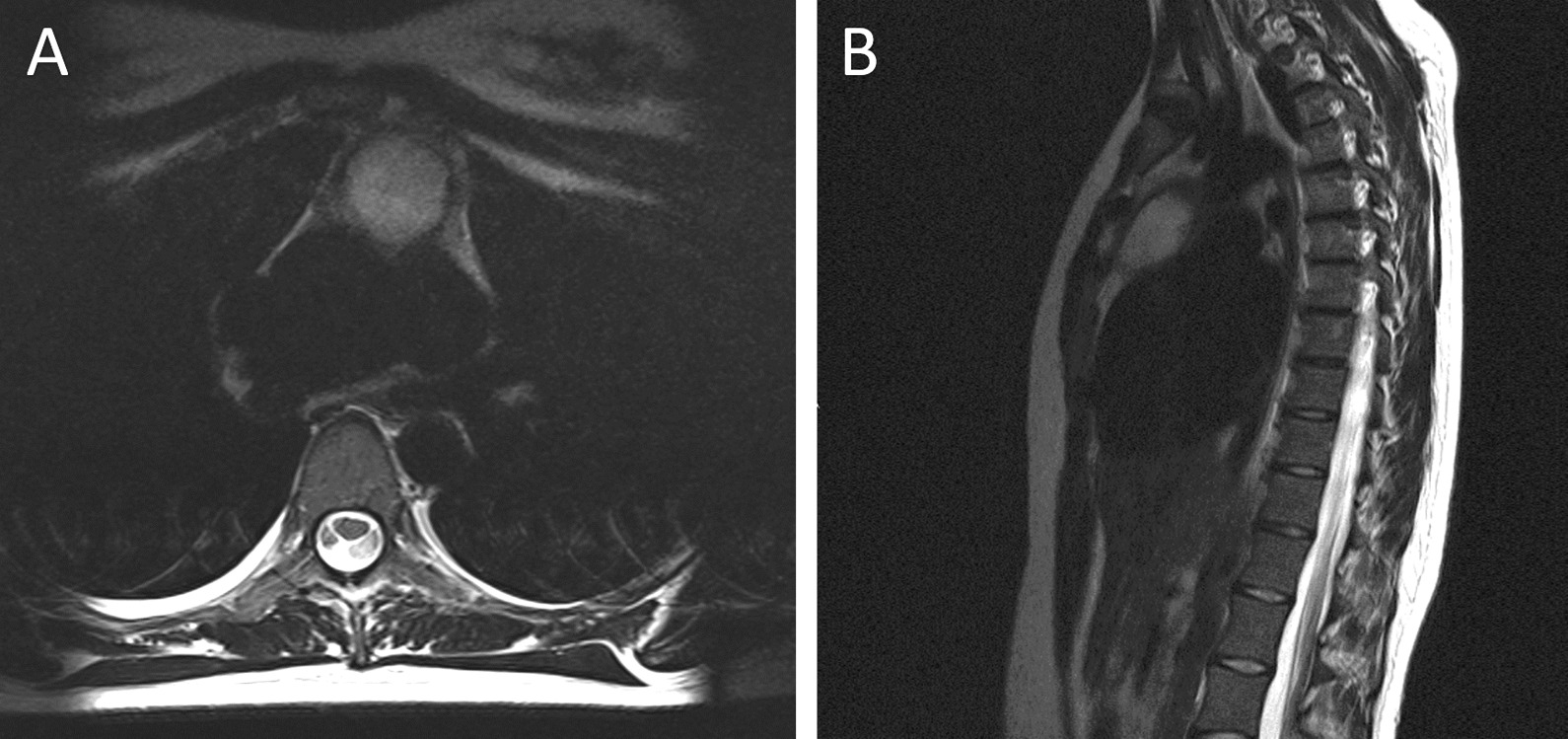
Magnetic resonance imaging (MRI) demonstrating anterior mediastinal mass (**A**) Transverse T2 image showing a heterogeneous anterior mediastinal mass. **B** Sagittal fat suppressed image demonstrating anterior mediastinal mass

**Fig. 2:**
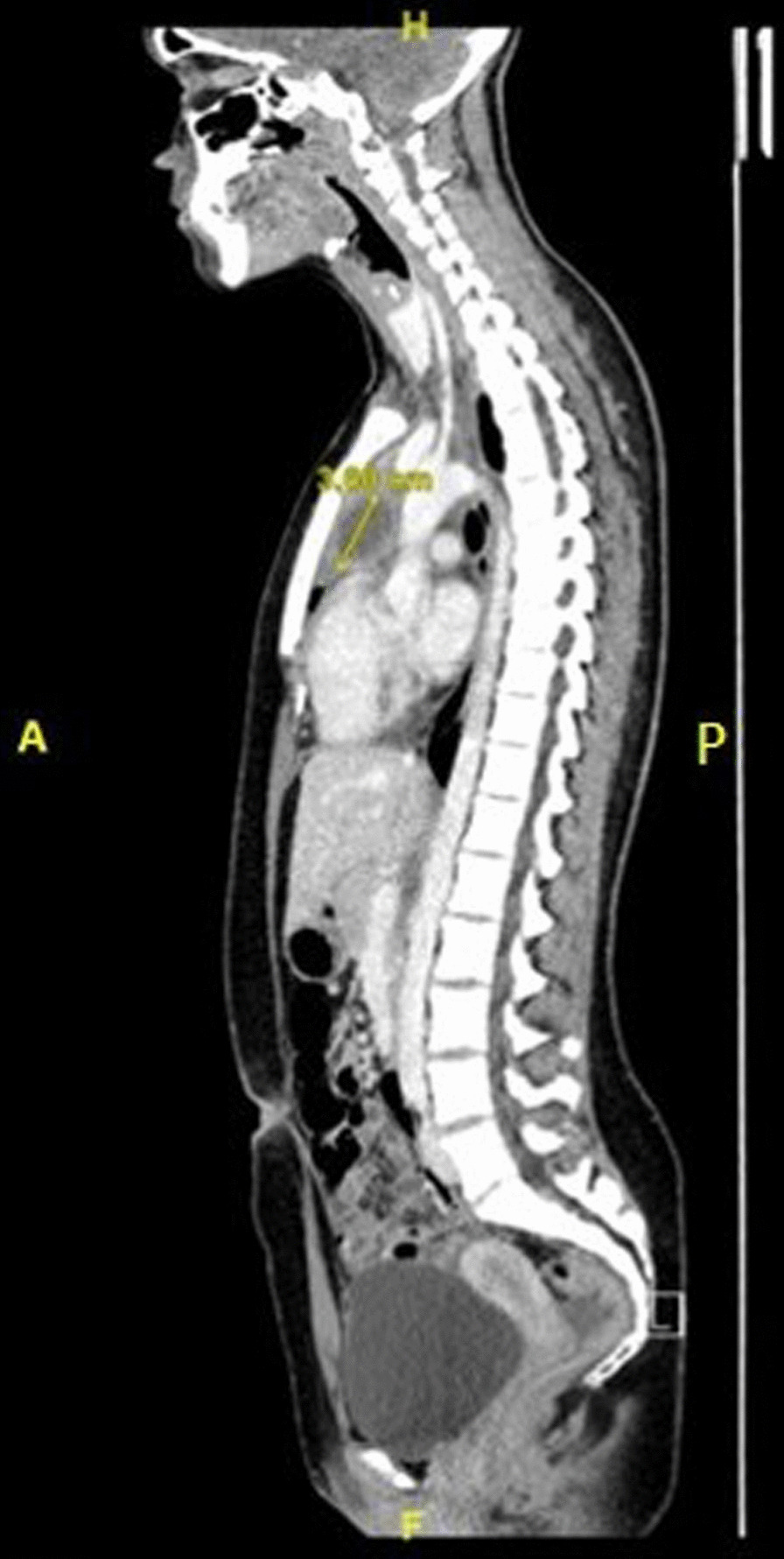
Sagittal plane of CT. This image highlights the presence of a 3.3cm × 3.8 cm cystic mass in the anterior mediastinum

Previous electromyography and nerve conduction studies in February 2019 reported no evidence of nerve lesions and no myopathic features at the time. In December 2019, neurologist arranged magnetic resonance imaging (MRI) of cervical spine, thoracic spine and brachial plexus which was initially reported as normal with respect to the spine and brachial plexus (Fig. 1). However, on further review of MRI at rheumatology multi-disciplinary team (MDT) meeting, an anterior mediastinal mass and lung shadowing was noted. Subsequent contrast-enhanced computed tomography (CT) of neck, thorax, abdomen and pelvis (Fig. 2) demonstrated a cystic anterior mediastinal mass measuring 3.3 × 3.8 cm thought to be a necrotic node which is typical of TB. There was evidence of consolidation in the right upper lobe with ‘tree-in-bud’ appearance. ‘Tree-in-bud’ refers to involvement of the smaller airways—a finding commonly seen in TB. The case was referred to chest MDT and was diagnosed as likely pulmonary TB. However, in view of the clinical-radiological correlation, she did not have symptoms of cough, fever or chest pain at this time.

Although blood tests for enzyme-linked immune absorbent spot assay (ELISpot®) were negative for TB and bronchoalveolar lavage (BAL) smear was negative for acid fast bacilli (AFB), she was treated empirically with the quadruple-antitubercular treatment Voractive® (combined tablet of rifampicin, isoniazid, pyrazinamide and ethambutol) with 10 mg pyridoxine. The plan was to continue treatment and follow-up with repeat imaging in 2 months.

Within 2 weeks of commencing treatment, the woman developed continuous cough unable to sleep lying flat. She was advised to take simple cough linctus and continue with the treatment. However, her cough worsened, with progressive retrosternal discomfort.

**Fig. 3:**
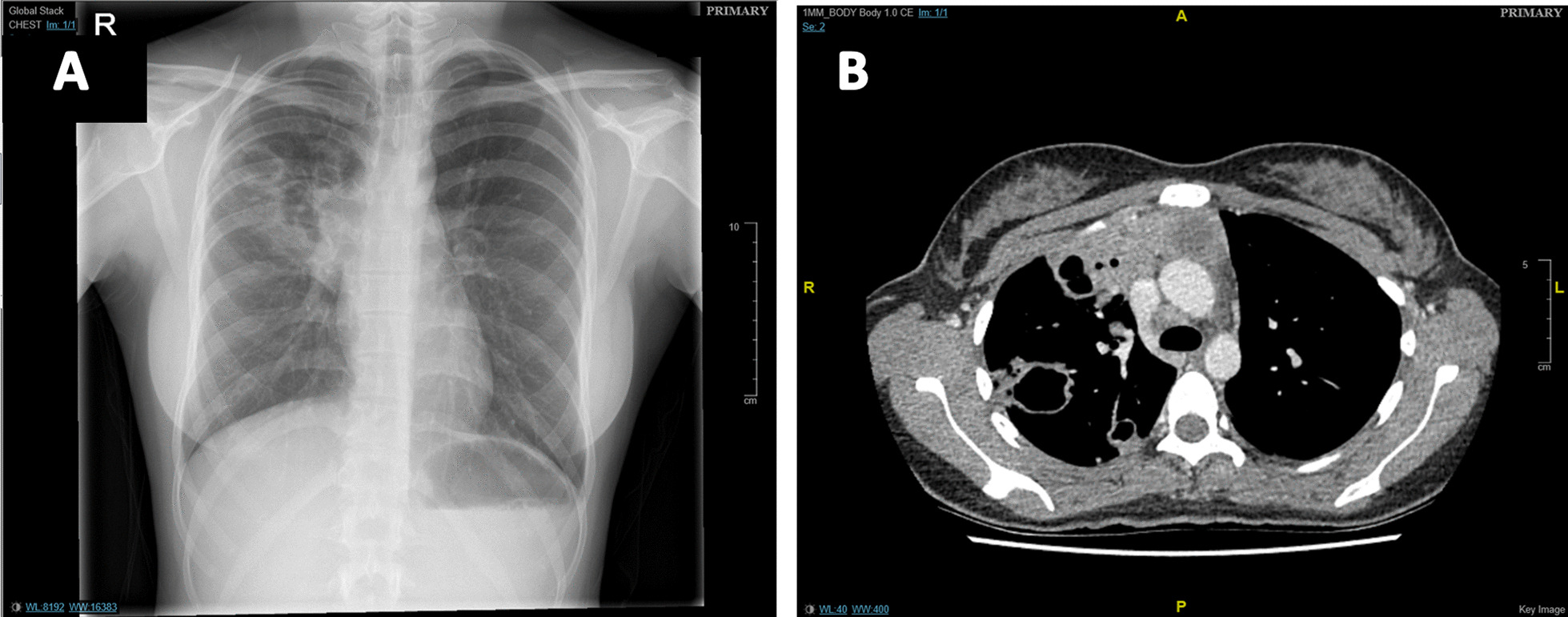
Imaging demonstrating cavitating lung lesions. **A** Chest X-ray demonstrating cavitating lesions in the right upper lobe. **B** Contrast-enhanced Chest CT scan showing cavitating lesions in the right lung with multiple nodular lesions bilaterally, along with an anterior mediastinal mass that is continuous with the posterior sternum

**Fig. 4:**
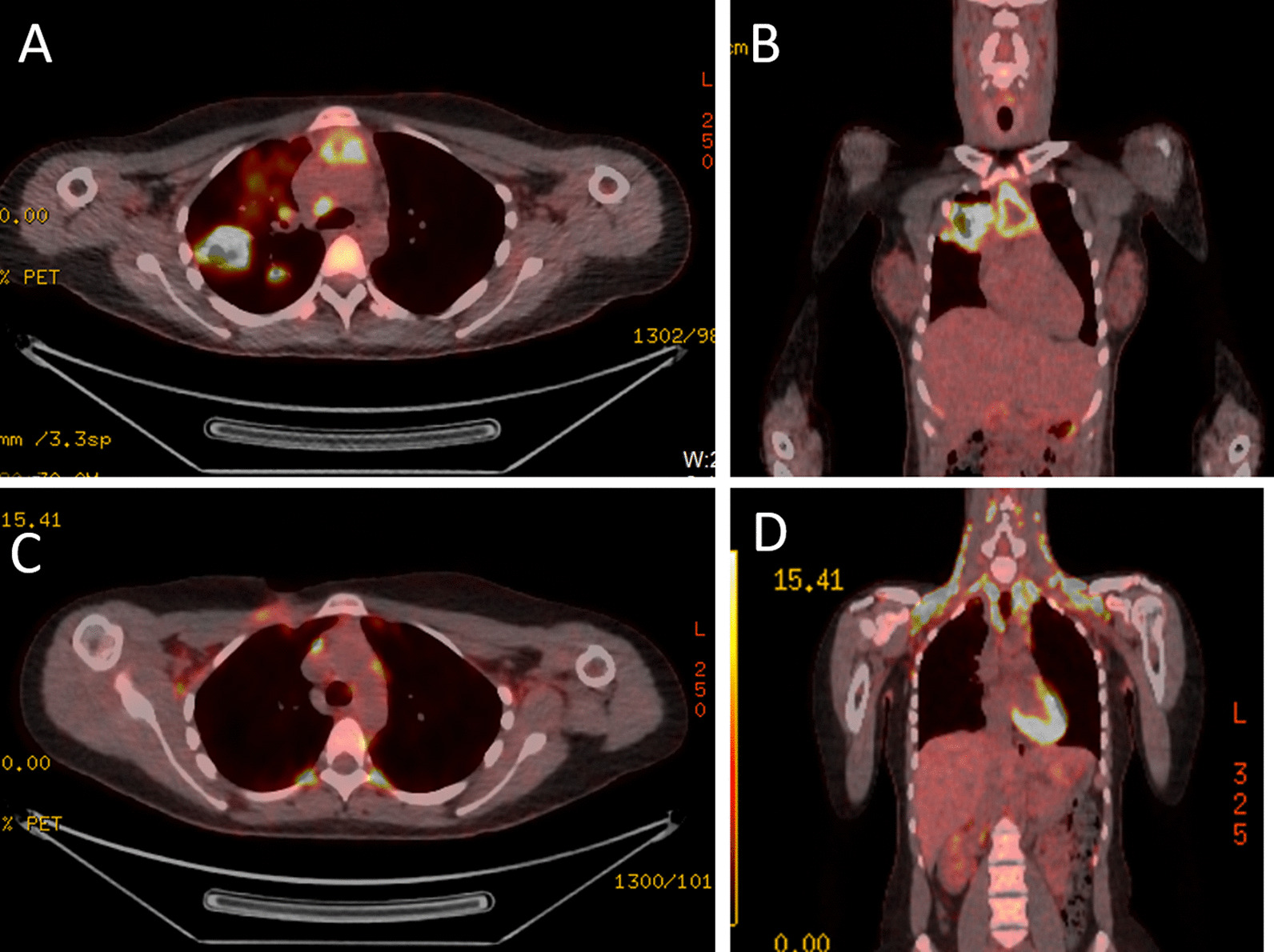
FDG-PET-CT scan images pre-and post-chemotherapy. **A** Pre-treatment axial section of FDG-PET-CT at mid-thoracic level showing metabolically active necrotic mediastinal lymph nodes and parenchymal lesions in the lungs. **B** Pre-treatment coronal section demonstrating metabolically active necrotic mediastinal and lung lesions. **C** Post-treatment axial images of FDG-PET-CT at mid-thoracic level showing complete resolution of the previously noted lung lesions with no significant residual activity. Background uptake seen on these images is due to physiological brown fat activity. **D** Post-treatment coronal section of FDG-PET-CT with no residual metabolically active disease. Supraclavicular fossa shows marked background of physiological brown fat activity

Her care was transferred to a hospital closer to her home. The four-drug regimen was continued for 2 months followed by 1 month of Rifinah® (rifampicin and isoniazid) with pyridoxine 10 mg. Her cough and chest discomfort did not improve. Repeat blood tests performed in April and May 2020 showed haemoglobin fell from 117 g/L to 101 g/L, WCC was persistently elevated at 25.1 × 10^9^/L and 20.2 × 10^9^/L with raised neutrophils of 22.49 × 10^9^/L and 19.62 × 10^9^/L respectively. Follow-up chest X-rays (CXR) and repeat CT scan demonstrated new cavitating lesions in the right lung with further enlargement of the mediastinal mass (Fig. 3). Sputum samples and repeat BAL sent for microscopy, culture and sensitivities (MC&S) and polymerase chain reaction (PCR) with GeneXpert® testing again yielded negative results for TB. A CT-guided needle biopsy of the lung and fluid aspiration of the mediastinal mass was performed. This yielded necrotic tissue on histology with negative AFB smear, negative GeneXpert® and microbiology. Following this, a whole-body fluorodeoxyglucose—positron emission tomography (FDG-PET)-CT scan was performed demonstrating metabolically active necrotic lesions in the mediastinum and lung parenchyma (Fig. 4A and 4B).

Histological analysis of mediastinotomy-derived lung biopsies demonstrated samples contained a nodular infiltrate of lymphoid cells admixed with bands of sclerotic tissue. The lymphoid tissue was polymorphous composed of small mature lymphoid cells admixed with histiocytes, eosinophils, neutrophils and some larger cells showing prominent nucleoli, binucleate forms and smudge cells. On immunohistochemistry, the large atypical cells were positive for CD30, CD15, MUM1, PAX5 and p53. They were negative for CD45, CD20, CD79a and CD3. The imaging and histological findings confirmed a diagnosis of Stage IVA classical Hodgkin’s lymphoma (nodular sclerosing subtype) in June 2020.

TB treatment was discontinued and the patient was urgently referred to the haemato-oncology team and commenced on the escalated BEACOP-DAC regimen (bleomycin, etoposide, doxorubicin, cyclophosphamide, vincristine, prednisolone and dacarbazine, with granulocyte colony stimulating factor). As time was of the essence, the option of cryopreservation of ova prior commencing chemotherapy was not possible. However, gonadotrophin releasing hormone (GnRH) analogue was offered. The FDG-PET-CT scan following the first two cycles demonstrated good response to chemotherapy (Deauville Score 3). Therefore, only two more cycles of escalated BEACOP-DAC were given. The post-chemotherapy FDG-PET-CT scan (Fig. 4C and 4D) showed resolution of disease, so no further treatment was indicated. At the time of writing, the patient was well and plan was to follow-up in haemato-oncology clinic.

## Discussion

**Fig. 5:**
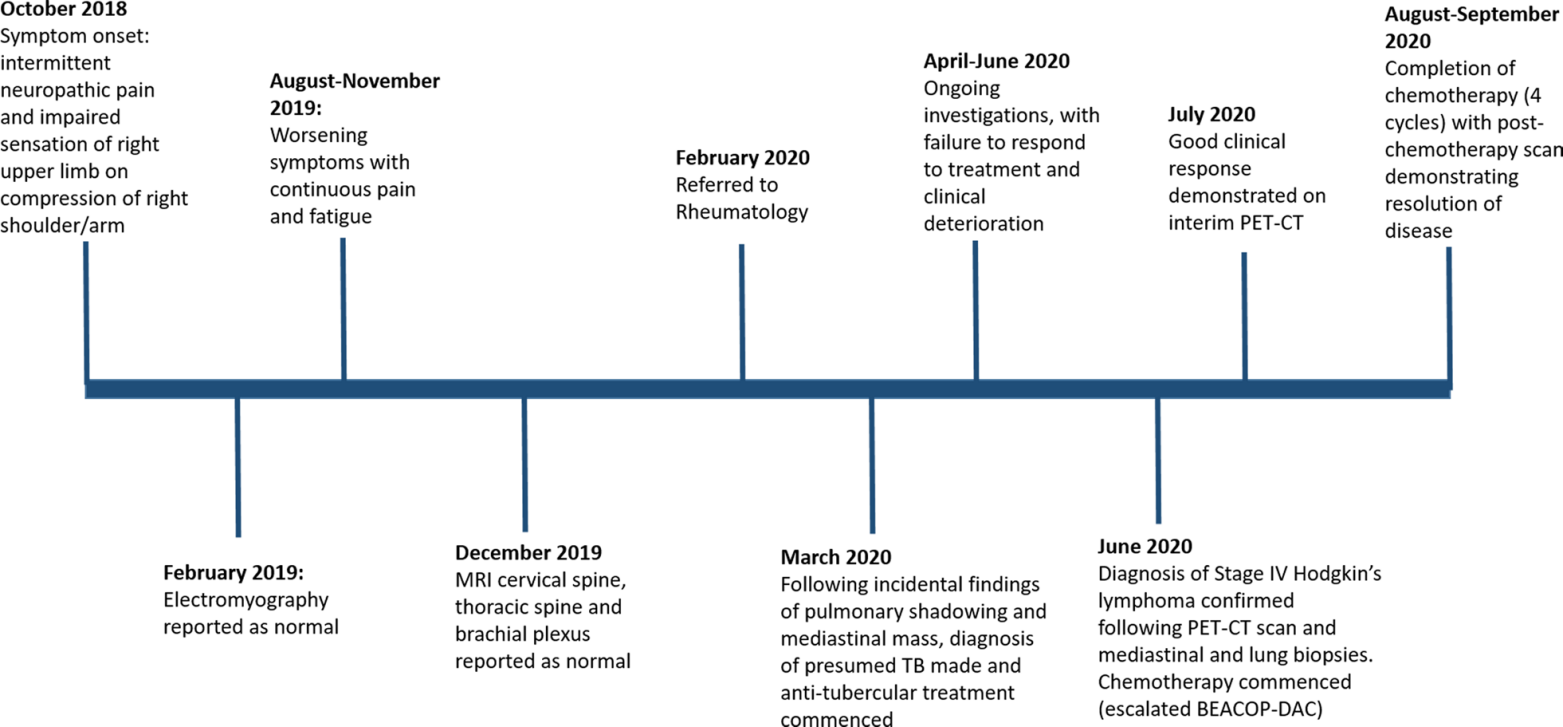
Timeline of case report. This timeline summarises the historical information and clinical interventions and management of this case

As demonstrated in the patient’s timeline (Fig. 5), the patient initially presented with possible neuropathic pain and was subsequently investigated with an MRI. Peripheral neuropathies can occur in 5-8% of patients with HL, which are mostly due to direct nerve compression by the tumour, deposition of immunoglobulins or secondary to treatment rather than true paraneoplastic phenomena [6]. However, paraneoplastic neuropathies can occur in B-cell lymphomas, the most common being Guillian-Barre syndrome or chronic inflammatory demyelinating polyneuropathy (CIDP) [6]*.* In this case, as MRI findings did not reveal any neurological abnormalities, it is unlikely this occurred.

On further review of the MRI, mediastinal and pulmonary pathology were incidentally found and were confirmed by contrast-enhanced CT scan. The top differential based on clinical-radiological diagnosis was TB.

TB is one of the leading causes of mortality worldwide, affecting approximately 10 million people globally, with highest incidence in South East Asia and Africa [7]. In the western world, the UK is a key hotspot with over 5000 cases per year [8]*.*

Pulmonary TB is divided into active and inactive forms. Imaging presentations of active TB include upper lobe consolidation with a ‘tree-in-bud' appearance as well as lung cavitation [9], which was seen in this case. TB can also present with lymphadenopathy within in the mediastinum, which is typically necrotic giving it a cystic appearance on CT. This was believed to explain the cystic anterior mediastinal mass in this case. However, the first CT report also suggested the mediastinal lesion could be a benign thymic cyst or thymoma. Based on epidemiology, the patient’s age and recent travel history, the diagnosis was in favour of TB and empirical treatment was commenced.

Where there is clinical suspicion of TB, diagnosis typically involves imaging (usually CXR or CT) and confirmation with isolation of *Mycobacterium tuberculosis* from multiple samples of bodily fluids or tissue using AFB smear tests, cultures and/or rapid nucleic acid amplification tests for example GeneXpert® [9, 10].

In this case, blood was tested for TB with ELIspot /T-SPOT*.TB®* blood test, and BAL smears were tested for AFB, which were all negative for TB prior commencing treatment. TB culture results received 6 weeks later were also negative.

Investigations such as blood tests and sputum samples are not always able to identify the presence of TB. Swai *et al.* [11] demonstrated the typical diagnostic work-up for smear-negative TB used in clinical practice had poor sensitivity and specificity of 38.1% and 74.5% respectively. Therefore, due to the importance of controlling disease spread, current guidelines from the National Institute of Clinical Excellence (NICE) recommend if active TB is suspected, anti-tubercular therapy should be started “without waiting for culture results” and courses should be continued with close monitoring of response despite negative results if TB is still considered likely [10]*.* In this case there was no history of recurrent cough, chest pain and temperature on presentation.

Despite continuing anti-TB treatment, the patient’s condition worsened. Repeated TB-focused tests were negative, and finally the diagnosis of HL was confirmed with an anterior mediastinal biopsy. We ask at what point should an alternative diagnosis be considered in a work-up for TB and when should we consider performing a biopsy—the definitive diagnostic test which can identify both TB and lymphoma?

Current NICE guidelines for TB do not clearly specify at what stages should alternative diagnoses be considered, nor do they provide details of key distinguishing factors that could assist in differentiating these conditions [10, 12].

The challenging nature of diagnosing lymphomas has been previously reported. Both TB and lymphoma can have similar non-specific systemic symptoms (fever, weight loss and night sweats), as well as lymphadenopathy, and granulomatous inflammation on cytology and histology [13]. The non-specific symptoms can often make it difficult for patients to seek medical help or prompt appropriate referral from primary care to diagnose patients in early stages of disease [14].

Raising awareness of key differences between TB and lymphoma could help improve diagnostic accuracy in earlier stages of disease.

Features suggesting non-TB pathology include: no known exposure to TB or previous TB infection, involvement of supraclavicular lymph nodes, negative first-line TB investigations [for example tuberculin skin test (TST), sputum samples for AFB smears], and lack of response to anti-tubercular treatment within the first 2 months [13]. However, TST can give false positive results where BCG vaccine is given, as in this case. Therefore, Elispot® test (T-SPOT.*TB®)* was performed.

A systematic review and meta-analysis demonstrated the T-SPOT*.TB®* test for blood samples had a greater pooled sensitivity of 81% (95% CI 78–84%) for all patients with TB (confirmed and non-confirmed by culture) and 92% (95% CI 90–93%) in those with culture-confirmed TB, compared to TST which only had sensitivity of 65% (95% CI 61–68%). However pooled specificity of the T-SPOT*.TB®* test was 59% (95% CI 56–62%), whilst TST was 75% (95% CI 72–78%) [15]. Comparatively, the GeneXpert® test using polymerase chain reaction (PCR) recommended by WHO and NICE have a sensitivity of 85% and specificity of 99% for detecting TB [16]*.* In our case, as the patient did not have symptoms of TB at presentation and ELIspot*®* and AFB smears of BAL were negative for TB, GeneXpert® should have been considered earlier to help rule out TB.

Imaging plays an integral role in diagnosing TB and lymphoma and is usually performed early in the diagnostic work up. Key differences in the anatomical distribution of lymphadenopathy and enhancement patterns for TB and lymphoma in contrast-enhanced CT imaging could help improve diagnostic accuracy. Lymphoma has a heterogeneous imaging appearance depending on the subtype. Nodular sclerosing classical HL is common in young females and often presents as an anterior mediastinal mass as seen in this case [17]. Other features include (multiple) nodules or masses which can cavitate, as seen in our case. Tang *et al.* [18] identified mediastinal lymphadenopathy in TB typically presents with peri-hilar, peripherally enhancing lymph nodes with a cystic or necrotic centre, whereas lymphomas have a more central, homogenous enhancement and most commonly affects superior lymph node regions that is para-aortic lymph nodes. Further studies could help by identifying imaging appearances which can discriminate between the common differentials.

In cases of smear-negative TB or suspected drug-resistant TB, the definitive diagnostic tests, such as CT imaging, molecular testing that is GeneXpert® and (immuno-)histopathological examination of biopsies, should be performed much earlier in the diagnostic work-up, preventing delay [9]. Immunohistological diagnosis of classical HL typically demonstrates characteristic Reed-Sternberg/Hodgkin cells positive for CD15 and CD30, with variable expression of CD20, but negative for CD79a, CD45 as seen in this case [19]. Although needle aspiration/biopsy is less invasive than excisional/incisional biopsy, several studies have demonstrated the sensitivity and specificity for diagnosing both TB and lymphoma are significantly higher with excision/incision biopsy, due to the larger sample and better quality of tissue obtained [15, 20]*.* For TB lymphadenitis, Knox *et al.* [21] found sensitivity of fine needle aspiration (FNA) specimens analysed via microscopy was 18%, cytology was 38% and culture improved this to 85%, but excision biopsy histology had highest sensitivity of 96%.

For lymphoma, Hehn *et al.* [20] found 72% of lymphoma patients who had an FNA required subsequent excision biopsy to confirm diagnosis. The preferred sample is an excisional or incisional biopsy to obtain adequate tissue for analysis [3, 22]. This is particularly important for classical HL, as the characteristic Reed-Sternberg cells within the inflammatory milieu are often few and sparse and may be missed with FNA or core-needle biopsy as the reactive inflammatory background can make up to 99% of the cell population [1, 9, 23]. Specificities could also be improved by processing histological samples for whole genome sequencing and immunohistochemistry with flow cytometry.

Other obligatory investigations include full blood count, ESR, CRP, lactate dehydrogenase, liver function and viral screening for HIV, Hepatitis B and C [3]*.* Our patient’s full blood count (FBC) showed elevated WCC, initially at 14.8 × 10^9^/L in February rising to 25.1 × 10^9^/L in April and remaining above 20 × 10^9^/L prior commencing chemotherapy. In active TB infection, the WCC is usually within normal range and may rise to approximately 13.5 × 10^9^/L [24]. Persistently elevated WCC above 15 × 10^9^/L should raise concerns about lymphoma [25]*.*

Findings from studies across the world highlight change is required to current guidelines to improve timing and accuracy of diagnosis of lymphoma. A study in South Africa investigating cases of lymphoma misdiagnosed as TB proposed a management algorithm where patients with presumed TB who are AFB smear-negative should initially be treated empirically with anti-tubercular medication, as per guidelines [5, 7, 10]. However, repeat testing and close monitoring must be implemented. If there is no improvement after 1 month of treatment and patient remains AFB smear-negative, then biopsies should be undertaken with histopathological analysis of TB as well as lymphoma and other possible pathology [5].

As shown in Table 1, although HL is very responsive to chemotherapy, it is an aggressive form of lymphoma and delay in diagnosis and treatment can affect prognosis [14]. Therefore, it is essential that a diagnostic approach involving earlier utilization of definitive tests described above as recommended by Puvaneswaran and Shoba [5] is implemented to update our guidelines for TB to prevent diagnostic delay of lymphoma.

**Table 1:** Lugano staging of classical Hodgyn lymphoma with 5-year net cancer survival rate

Lugano staging classification	Definition	5-year net cancer survival rate (%)
I	I-Single lymph node or one group of lymph nodes or one lymphatic organ affected IE-one extra-nodal site in the absence of lymph-node involvement	90
II	II-Two or more groups of lymph nodes affected on the same side of diaphragam IIE-Contiguous extra nodal extension from a nodal site with or without other groups of lymph nodes on the same side of diaphragm (IIE)	90
III	Lymph nodes are affected on the same side of the diaphragam III (1)-spleen/splenic nodes involved III (2)-para-aortic, iliac, inguinal or mesenteric nodes involved	80
IV	Diffuse or disseminated disease with one or more extranodal sites affected including liver, lungs, bone OR extra-nodal organs affected without associated lymph node involvement	70

Limited disease (Lugano Stage I and II) has the greatest prognosis and cure rates with 90% 5-year survival, whilst advanced disease (Lugano Stage III and IV) has poorer prognosis, with 5-year survival at 70–80% [3, 22, 26–28]

## Conclusions

Despite advances in knowledge, technology and medical practice, the diagnostic dilemma of TB and lymphoma still affects patients across the world. HL is a malignancy typically seen in children and young adults and should be considered as a differential in patients with lymphadenopathy. Imaging features can be difficult to differentiate from other presentations and correlation with clinical and biochemical findings is essential. Diagnostic difficulties can lead to increased number of investigations and delay in diagnosis and treatment of lymphoma and improve patient prognosis.

Existing clinical guidelines used in both primary care and in hospitals should be updated and refined to improve diagnosis at earlier stages of disease. In smear-negative TB cases, definitive diagnostic tests that is molecular testing with GeneXpert® and excisional/incisional biopsies could be considered earlier in the diagnostic work-up to distinguish between TB or lymphoma. Further research is required to develop better non-invasive diagnostic methods and better understand and address factors affecting diagnostic delay of lymphoma to improve patient care.

## Data Availability

Not applicable.

## Abbreviations

AFB: Acid fast bacilli
BAL: Bronchoalveolar lavage
CT: Computed tomography
CXR: Chest x-ray
FDG-PET-CT: Fluorodeoxyglucose positron emission tomography-computed tomography
FNA: Fine needle aspiration
HL: Hodgkin’s Lymphoma
MDT: Multidisciplinary team
MRI: Magnetic resonance imaging
TB: Tuberculosis
